# Treatment-related complications of radiation therapy after radical prostatectomy: comparative effectiveness of intensity-modulated versus conformal radiation therapy

**DOI:** 10.1002/cam4.205

**Published:** 2014-02-12

**Authors:** Edwin F Crandley, Sarah E Hegarty, Terry Hyslop, David D Wilson, Adam P Dicker, Timothy N Showalter

**Affiliations:** 1 Department of Radiation Oncology, University of VirginiaCharlottesville, Virginia; 2 Division of Biostatistics, Thomas Jefferson UniversityPhiladelphia, Pennsylvania; 3 Department of Radiation Oncology, Thomas Jefferson UniversityPhiladelphia, Pennsylvania

**Keywords:** Comparative effectiveness research, Intensity-modulated radiotherapy, prostate cancer, prostatectomy, radiation therapy complications

## Abstract

Intensity-modulated radiation therapy (IMRT) is frequently utilized after prostatectomy without strong evidence for an improvement in outcomes compared to conformal radiation therapy (RT). We analyzed a large group of patients treated with RT after radical prostatectomy (RP) to compare complications after IMRT and CRT. The Surveillance, Epidemiology and End Results (SEER)-Medicare database was queried to identify male Medicare beneficiaries aged 66 years or older who underwent prostatectomy with 1+ adverse pathologic features and received postprostatectomy RT between 1995 and 2007. Chi-square test was used to compare baseline characteristics between the treatment groups. First complication events, based upon administrative procedure or diagnosis codes occurring >1 year after start of RT, were compared for IMRT versus CRT groups. Propensity score adjustment was performed to adjust for potential confounders. Multivariable Cox proportional hazards models of time to first complication were performed. A total of 1686 patients were identified who received RT after RP (IMRT = 634, CRT = 1052). Patients treated with IMRT were more likely to be diagnosed after 2004 (*P* < 0.001), have minimally invasive prostatectomy (*P* < 0.001) and have positive margins (*P* = 0.019). IMRT use increased over time. After propensity score adjustment, IMRT was associated with lower rate of gastrointestinal (GI) complications, and higher rate of genitourinary-incontinence complications, compared to CRT. The observed outcomes after IMRT must be considered when determining the optimal approach for postprostatectomy RT and warrant additional study.

## Introduction

Prospective randomized trials have demonstrated an improvement in biochemical progression-free survival 1–3, distant metastasis-free survival 4, and overall survival 4 with the addition of adjuvant radiation therapy (RT) to the prostate fossa after radical prostatectomy (RP) for men with high-risk pathological features (positive surgical margins, extracapsular extension, and/or seminal vesicle invasion). However, many clinicians prefer delayed salvage RT for patients selected based upon prostate serum antigen (PSA)-defined recurrence, rather than immediate treatment, due to concerns about overtreatment and complications with adjuvant radiation therapy (ART), as well as published evidence that supports the effectiveness of salvage RT 5,6. Retrospective data have demonstrated that salvage RT for PSA failure after RP improves prostate cancer-specific mortality 7.

The prostate bed is the most common site of failure after RP 8, and the volume targeted by postprostatectomy RT encompasses the prostate bed, including the vesicourethral anastomosis and seminal vesicle remnant along with added margin for setup error 9. As the prostate bed target volume is adjacent to normal tissue structures, including bladder and rectum, advanced delivery approaches have been investigated. Dosimetric comparisons of intensity-modulated RT (IMRT) to conformal RT (CRT) techniques have shown that IMRT treatment planning reduces the volume of bladder and rectum exposed to high RT doses and allows for dose escalation to the target volume without exceeding bladder and rectum constraints 10,11.

IMRT has been adopted widely in the United States, with nearly 90% of radiation oncologists reporting the use of IMRT for postprostatectomy treatment 6, consistent with an overall trend of increasing use of IMRT for prostate cancer over the past decade 12. However, there is scant clinical evidence to show that the dosimetric superiority of IMRT over CRT translates into more effective treatments for prostate cancer patients, and IMRT has not been compared to CRT in randomized controlled trials. Some small studies have provided encouraging early clinical results of postprostatectomy IMRT 13,14, but the increased expense of IMRT over CRT 10,12 warrants more and high-quality data to evaluate the comparative effectiveness of these techniques in this setting.

In this report, we evaluate the comparative effectiveness of IMRT versus CRT for adjuvant and salvage RT after RP among a cohort of elderly patients who qualified for ART based upon the presence of adverse pathological features in the surgical specimen. As the rationale for IMRT over CRT is primarily the reduced risk of complications through improved normal tissue sparing from high RT doses 15,16, we compared the incidence of genitourinary (GU), gastrointestinal (GI), and sexual complications between these two techniques.

## Methods and Materials

### Study design

This was an observational cohort study of complications after IMRT or CRT using the linked Surveillance, Epidemiology and End Results (SEER)-Medicare database, a research resource that links the SEER tumor registry with Medicare administrative claims 17. From among the 523,153 prostate cancer cases recorded in the SEER registry during 1992–2008, we identified patients who received RP, had one or more adverse pathologic feature (seminal vesicle invasion, extracapsular extension, positive surgical margins), and who received postoperative RT between the years of 1995 and 2007. To increase the accuracy of measured health claims, only men aged 66 years or older at time of diagnosis were included to ensure that 1 year of data were available prior to diagnosis; men who were also enrolled in a health maintenance organization during the study were excluded; and men who were not continually enrolled in both Medicare Parts A and B starting 1 year prior to diagnosis were excluded. Additional exclusion criteria included involved lymph nodes, treatment with brachytherapy, and treatment with proton beam therapy (Fig. S1). Based upon the SEER data variables, a cohort of 6345 subjects was identified, and subsequent exclusions were based upon Medicare data elements. Delivered treatments (RP, IMRT, CRT) were identified based upon Current Procedural Terminology (CPT), Healthcare Common Procedure Coding System (HCPCS), and associated International Classification of Diseases (ICD) codes (Table S1). These administrative claims codes were adapted based upon review of several prior published reports 15,16,18,19. The final cohort comprised only men who met eligibility criteria for ART after RP, and included 1052 subjects who received CRT and 634 subjects who received IMRT after RP (Fig. S1).

### Study variables

Rates of first complication events occurring 1 year or more after start of RT were compared between the treatment groups. First complication events were defined based upon HCPCS/CPT-4 procedure codes and ICD-9 diagnosis codes (Table S1). Complication events for analysis were limited to those occurring 1 year or more after start of RT as the focus of this study was a comparison of late treatment-related toxicity. The primary outcome was rate of GI complications after RT. Secondary outcomes were rates of GU incontinence, GU nonincontinence, and erectile dysfunction (ED). Baseline characteristics obtained from the SEER-Medicare database were race, marital status, education level, income, population density of place of residence, region of place of residence, year of diagnosis, pathologic stage, Gleason score, pathologic margin status, age at diagnosis, comorbidities, use of androgen deprivation therapy (ADT), and surgical technique (minimally invasive or open retropubic RP). RT dose data or other technical details are not available in the SEER-Medicare database.

### Statistical analysis

Baseline characteristics between the IMRT and CRT groups were compared using the chi-square test. A propensity score was calculated for each person using logistic regression to model the probability of treatment. The following variables were included in the propensity score model: race, hispanic origin, marital status, census-tract% high school completion, census-tract median income, population density, SEER region, year of diagnosis, pT stage, Gleason score, margin status, age at diagnosis, comorbidity score, ADT receipt, and surgery type. A propensity score weight was calculated as the inverse predicted probability of being in one's treatment group; this weight was then adjusted by the relative sample size of each treatment group. First complication events (based on either procedure or diagnosis code) were reported in events/100 person years and adjustment for potential confounders was performed by propensity score weighting 20. 95% confidence intervals of adjusted rate ratios were calculated by weighted Poisson regression.

Multivariate Cox proportional hazards models of time to first complication (based on procedure codes) were performed for each class of complications. Covariates forced into all models were pathologic T stage (T2, T3a, T3b), Gleason score (≤7, 8+), surgical margins (involved, not involved), age at diagnosis (66–69, 70–74, 75–79, 80+), surgery (open vs. minimally invasive), and ADT use (yes, no), and year of diagnosis (1995–1999, 2000–2004, 2005–2007). The remaining variables from Table1 were considered for multivariable models if they had a level of significance <0.3 on univariable analysis. The covariate with the largest *P* value was then removed from the model so long as the parameter estimate of treatment effect was not changed by more than 20%. This process was repeated until all covariates had a *P* value of less than 0.1. There was one exception to the 20% change rule utilized in the urinary-nonincontinence model. In this model, several removed covariates impacted the very small treatment effects by >20%; however, the resulting estimates of difference between RT types were still near zero. Thus, the removed covariates were left out of the final models presented in Tables 3–6. Hazard ratios were calculated with 95% confidence intervals and *P* values were considered significant if <0.05. All Cox models were weighted by the adjusted propensity.

**Table 1:** Baseline characteristics of the CRT and IMRT cohorts.

	CRT (*n* = 1052)	IMRT^1^ (*n* = 634)	*P*-value (*χ*^2^)
*Demographic factors*			
Race			
White	940 (89.35)	566 (89.27)	0.882
Black	57 (5.42)	32 (5.05)	
Asian/Pacific Islander/other	55 (5.23)	36 (5.68)	
Hispanic			
Non-Hispanic	1000 (95.06)	589 (92.90)	0.066
Hispanic	52 (4.94)	45 (7.10)	
Marital status			
Not married	136 (12.93)	79 (12.46)	0.958
Married	892 (84.79)	540 (85.17)	
Unknown	24 (2.28)	15 (2.37)	
Education^2^			
<75%	153 (14.54)	120 (18.93)	0.028
75–84.99%	242 (23.00)	117 (18.45)	
85–89.99%	213 (20.25)	120 (18.93)	
90%+	444 (42.21)	277 (43.69)	
Income^2^			
<35K	188 (17.87)	119 (18.77)	0.027
35–44K	241 (22.91)	115 (18.14)	
45–59K	284 (27.00)	158 (24.92)	
60K+	339 (32.22)	242 (38.17)	
Population density			
Urban	1029 (97.81)	626 (98.74)	0.171
Rural	23 (2.19)	8 (1.26)	
Region			
West	628 (59.70)	419 (66.09)	<0.001
Midwest	223 (21.20)	56 (8.83)	
Northeast	90 (8.56)	84 (13.25)	
South	111 (10.55)	75 (11.83)	
Year of diagnosis			
1995–1999	434 (41.25)	30 (4.73)	<0.001
2000–2004	522 (49.62)	260 (41.01)	
2005–2007	96 (9.13)	344 (54.26)	
*Tumor-related factors*			
pT			
T2	230 (21.86)	156 (24.61)	0.426
T3a	550 (52.28)	318 (50.16)	
T3b	272 (25.86)	160 (25.24)	
Gleason score			
≤7	541 (51.43)	346 (54.57)	0.210
8+	511 (48.57)	288 (45.43)	
Margins			
Uninvolved	576 (54.75)	310 (48.90)	0.020
Involved	476 (45.25)	324 (51.10)	
Age at diagnosis			
66–69	594 (56.46)	367 (57.89)	0.713
70–74	375 (35.65)	221 (34.86)	
75–79	78 (7.41)	41 (6.47)	
80+	5 (0.48)	5 (0.79)	
Comorbidity			
0	697 (66.25)	411 (64.83)	0.746
1	253 (24.05)	163 (25.71)	
2+	102 (9.70)	60 (9.46)	
*Treatment factors*			
ADT			
No	470 (44.68)	270 (42.59)	0.402
Yes	582 (55.32)	364 (57.41)	
Surgery			
ORP	1017 (96.67)	480 (75.71)	<0.001
MIRP	35 (3.33)	154 (24.29)	

CRT, conformal radiation therapy; IMRT, intensity-modulated radiation therapy; ADT, androgen deprivation therapy; ORP, open radical prostatectomy; MIRP, minimally invasive radical prostatectomy.

1 Those with both IMRT and CRT (*n* = 215) are considered IMRT.

2 Census level variables.

## Results

### Baseline patient characteristics

Overall, 1686 patients were identified who met the inclusion criteria, including 1052 who were treated with CRT and 634 were treated with IMRT. The median follow up was 5.7 years for the CRT group and 2 years for IMRT group. The median time to post-RP RT was 7.5 months for the CRT group and 10.2 months for the IMRT group. The IMRT and CRT groups differed according to region, year of diagnosis, education level, and income level (Table 1). There was a trend toward increased utilization of IMRT over time. Patients treated with IMRT were also more likely both to have positive surgical margins and to have received a minimally invasive (vs. open) RP (Table 1).

### Adjusted complication rates

Rates of first complication, according to class of complication, were adjusted based upon propensity score using Table 1 variables, and are presented in units of events per 100 person years with rate ratios and 95% confidence intervals (Table 2). Additional data regarding time interval between RT and complications are presented in Table S2. Patients treated with IMRT had a lower rate of GI complications but a higher rate of GU incontinence complications based on procedure codes when compared to the CRT group. There was no difference in the rate of ED or GU nonincontinence complications between the treatment groups (Table 2). Unadjusted rates of first complications are presented in Table S3.

**Table 2:** Propensity score-adjusted complication rates, defined by procedure and diagnosis codes, listed according to class of complications.

Complications by class	CRT (*n* = 1052)	IMRT (*n* = 634)	IMRT vs. CRT comparison
	Events/100 person years	Events/100 person years	Rate ratio (95% CI)
Erectile dysfunction			
Procedure	0.82	0.50	0.61 (0.29, 1.29)
Diagnosis	6.71	7.24	1.08 (0.86, 1.35)
Gastrointestinal			
Procedure	13.16	8.80	0.67 (0.55, 0.81)
Diagnosis	11.05	9.93	0.90 (0.74, 1.09)
Urinary–incontinence			
Procedure	4.46	8.06	1.81 (1.44, 2.27)
Diagnosis	7.22	9.46	1.31 (1.07, 1.60)
Urinary-nonincontinence			
Procedure	3.21	2.86	0.89 (0.64, 1.24)
Diagnosis	6.45	7.38	1.14 (0.92, 1.43)

CRT, conformal radiation therapy; IMRT, intensity-modulated radiation therapy.

### Multivariable analysis of time to first complication

Cox proportional hazards models of the time to first GI, urinary-incontinence, urinary-nonincontinence, and ED complications (procedures) are presented in Tables 3, 4, 5 and 6, respectively. On multivariable analysis, use of IMRT was associated with a lower rate of GI, and higher rate of urinary-incontinence procedure events compared to CRT.

**Table 3:** Propensity-score-weighted cox proportional hazards model: time to first GI complication (using procedure codes).

Parameter	Hazard ratio	95% Confidence interval	*P*	Overall *P*
RT type				
IMRT vs. CRT	0.68	(0.56, 0.83)	<0.001	<0.001
pT stage				
T3a vs. T2	0.96	(0.75, 1.22)	0.711	0.744
T3b vs. T2	0.88	(0.63, 1.24)	0.463	
Gleason				
8+ vs. <7	1.04	(0.88, 1.23)	0.657	0.657
Margins				
Involved vs. uninvolved	1.05	(0.83, 1.32)	0.712	0.712
Age at diagnosis				
70–74 vs. 66–69	0.99	(0.83, 1.18)	0.894	0.894
75–79 vs. 66–69	0.89	(0.62, 1.28)	0.531	
80+ vs. 66–69	0.75	(0.24, 2.39)	0.625	
Surgery				
MIRP vs. ORP	0.94	(0.63, 1.43)	0.786	0.786
ADT				
Yes vs. no	1.03	(0.86, 1.22)	0.766	0.766
Year of diagnosis				
2000–2004 vs. 1995–1999	1.23	(1.01, 1.49)	0.042	0.020
2005–2007 vs. 1995–1999	0.86	(0.61, 1.21)	0.395	
Race				
Black vs. white	1.48	(1.07, 2.06)	0.019	0.006
Other vs. white	1.53	(1.07, 2.18)	0.019	
Hispanic origin				
Yes vs. no	0.59	(0.37, 0.93)	0.024	0.024
Median household income				
<35K vs. 35–44K	0.92	(0.69, 1.22)	0.544	0.015
45–59K vs. 35–44K	1.19	(0.93, 1.52)	0.165	
60K+ vs. 35–44K	1.35	(1.07, 1.70)	0.011	
Region				
Midwest vs. west	1.27	(1.00, 1.60)	0.047	0.049
Northeast vs. west	1.26	(0.95, 1.66)	0.106	
South vs. west	1.35	(1.03, 1.77)	0.031	

GI, gastrointestinal; RT, radiation therapy; CRT, conformal radiation therapy; IMRT, intensity-modulated radiation therapy; ADT, androgen deprivation therapy; ORP, open radical prostatectomy; MIRP, minimally invasive radical prostatectomy.

Proportionality assumption test, *P* = 0.077.

**Table 4:** Propensity-score-weighted cox proportional hazards model: time to first UI complication (using procedure codes).

Parameter	Hazard ratio	95% Confidence interval	*P*	Overall *P*
RT type				
IMRT vs CRT	1.90	(1.49, 2.42)	<0.001	<0.001
pT stage				
T3a vs. T2	0.71	(0.51, 0.99)	0.044	0.074
T3b vs. T2	0.60	(0.38, 0.94)	0.028	
Gleason				
8+ vs. <7	0.89	(0.71, 1.12)	0.321	0.321
Margins				
Involved vs. uninvolved	0.85	(0.62, 1.17)	0.316	0.316
Age at diagnosis				
70–74 vs. 66–69	1.48	(1.17, 1.87)	0.001	0.007
75–79 vs. 66–69	1.60	(1.03, 2.48)	0.038	
80+ vs. 66–69	1.07	(0.23, 4.91)	0.928	
Surgery				
MIRP vs. ORP	0.74	(0.42, 1.32)	0.312	0.312
ADT				
Yes vs. no	1.04	(0.83, 1.31)	0.739	0.739
Year of diagnosis				
2000–2004 vs. 1995–1999	0.97	(0.73, 1.27)	0.809	0.762
2005–2007 vs. 1995–1999	1.11	(0.73, 1.70)	0.615	
Hispanic origin				
Yes vs. no	1.75	(1.15, 2.67)	0.009	0.009
Region				
Midwest vs. west	0.84	(0.60, 1.19)	0.327	0.012
Northeast vs. west	1.11	(0.75, 1.64)	0.590	
South vs. west	1.64	(1.18, 2.29)	0.004	

UI, urinary incontinence; RT, radiation therapy; CRT, conformal radiation therapy; IMRT, intensity-modulated radiation therapy; ADT, androgen deprivation therapy; ORP, open radical prostatectomy; MIRP, minimally invasive radical prostatectomy.

Proportionality assumption test, *P* = 0.079.

**Table 5:** Propensity-score-weighted cox proportional hazards model: time to first UN complication (using procedure codes).

Parameter	Hazard ratio	95% Confidence interval	*P*	Overall *P*
RT type				
IMRT vs CRT	1.02	(0.71, 1.48)	0.912	0.912
pT stage				
T3a vs. T2	1.16	(0.75, 1.81)	0.502	0.669
T3b vs. T2	1.31	(0.73, 2.36)	0.370	
Gleason				
8+ vs. <7	0.81	(0.61, 1.09)	0.172	0.172
Margins				
Involved vs. uninvolved	0.96	(0.64, 1.43)	0.828	0.828
Age at diagnosis				
70–74 vs. 66–69	1.43	(1.06, 1.93)	0.021	0.074
75–79 vs. 66–69	1.36	(0.78, 2.38)	0.283	
80+ vs. 66–69	2.77	(0.68, 11.23)	0.154	
Surgery				
MIRP vs. ORP	0.48	(0.20, 1.14)	0.095	0.095
ADT				
Yes vs. no	1.43	(1.05, 1.94)	0.022	0.022
Year of diagnosis				
2000–2004 vs. 1995–1999	0.89	(0.62, 1.27)	0.523	0.814
2005–2007 vs. 1995–1999	0.90	(0.50, 1.61)	0.714	
Marital status				
Not married vs. married	1.50	(1.02, 2.21)	0.038	0.099
Unknown vs. married	0.75	(0.22, 2.54)	0.640	
Median household income				
<35K vs. 35–44K	1.44	(0.89, 2.31)	0.137	0.067
45–59K vs. 35–44K	1.14	(0.72, 1.81)	0.584	
60K+ vs. 35–44K	1.65	(1.08, 2.51)	0.019	
Region				
Midwest vs. west	0.60	(0.37, 0.97)	0.036	0.008
Northeast vs. west	0.71	(0.42, 1.21)	0.209	
South vs. west	1.52	(0.98, 2.34)	0.059	
Time from RP to RT				
90–179 days vs. <90 days	1.07	(0.70, 1.62)	0.761	0.087
180–359 days vs. <90 days	0.97	(0.61, 1.55)	0.913	
360–899 days vs. <90 days	0.66	(0.40, 1.08)	0.097	
900+ days vs. <90 days	0.60	(0.35, 1.02)	0.059	

UN, urinary non-incontinence; RT, radiation therapy; CRT, conformal radiation therapy; IMRT, intensity-modulated radiation therapy; ADT, androgen deprivation therapy; RP, radical prostatectomy; ORP, open radical prostatectomy; MIRP, minimally invasive radical prostatectomy.

Proportionality assumption test, *P* = 0.190.

**Table 6:** Propensity-score-weighted cox proportional hazards model: time to first ED complication (using procedure codes).

Parameter	Hazard ratio	95% Confidence interval	*P*	Overall *P*
RT type				
IMRT vs CRT	0.71	(0.32, 1.57)	0.395	0.395
pT stage				
T3a vs. T2	2.36	(0.89, 6.22)	0.084	0.060
T3b vs. T2	4.63	(1.30, 16.53)	0.018	
Gleason				
8+ vs. <7	1.15	(0.63, 2.10)	0.639	0.639
Margins				
Involved vs. uninvolved	1.35	(0.60, 3.06)	0.468	0.468
Age at diagnosis				
70–74 vs. 66–69	0.65	(0.35, 1.22)	0.179	0.196
75+ vs. 66–69	0.32	(0.06, 1.67)	0.177	
Surgery				
MIRP vs. ORP	3.74	(1.59, 8.80)	0.003	0.003
ADT				
Yes vs. no	0.58	(0.32, 1.06)	0.076	0.076
Year of diagnosis				
2000–2004 vs. 1995–1999	0.86	(0.41, 1.83)	0.697	0.898
2005–2007 vs. 1995–1999	0.80	(0.29, 2.21)	0.665	
Hispanic origin				
Yes vs. no	3.00	(1.19, 7.59)	0.020	0.020
At least HS education (zip)				
75–85% vs. <75%	1.58	(0.61, 4.13)	0.347	0.076
85–90% vs. <75%	2.16	(0.83, 5.64)	0.117	
90%+ vs. <75%	0.86	(0.33, 2.28)	0.764	
Time from RP to RT				
90–179 days vs. <90 days	3.33	(1.30, 8.57)	0.012	0.006
180–359 days vs. <90 days	2.21	(0.79, 6.17)	0.130	
360–899 days vs. <90 days	1.22	(0.38, 3.93)	0.744	
900+ days vs. <90 days	0.12	(0.01, 1.57)	0.106	

ED, erectile dysfunction; RT, radiation therapy; CRT, conformal radiation therapy; IMRT, intensity-modulated radiation therapy; ADT, androgen deprivation therapy; RP, radical prostatectomy; ORP, open radical prostatectomy; MIRP, minimally invasive radical prostatectomy; HS, high school.

Proportionality assumption test, *P* = 0.674.

## Discussion

In this population-based cohort study, the use of IMRT for postprostatectomy RT was associated with a decreased rate of GI complications when compared to CRT. The rate of urinary-incontinence complications was higher for the IMRT group, and there was no difference between the treatment groups in the two other classes of complications evaluated: urinary-nonincontinence and ED. The delivery of RT within 3 months after RP was not associated with increased risk of complications in this cohort, which has potential implications for clinical decisions regarding early versus delayed RT after RP.

The observed decreased risk of GI complications associated with the use of IMRT is not unexpected, as dosimetric studies have demonstrated that IMRT decreases the volume of rectum receiving high doses 10,11. This is consistent with findings from population-based comparative effectiveness analyses in intact prostate gland RT where IMRT resulted in lower rates of GI toxicity 15,16. Additionally, single institution retrospective data of salvage RT suggested that IMRT was associated with a decreased rate of grade 2+ GI toxicity at 5 years (1.9% vs. 10.2%) 21.

Perhaps most notably, however, our finding in this study concerning GI complications is in disagreement with the findings of Goldin and colleagues, who identified no differences in GI, GU, or sexual complications in their comparison of IMRT and CRT in a similar cohort of prostate cancer patients from the SEER-Medicare database 22. This study cohort differs from the Goldin et al. study by including subjects from a longer time period, including RT delivered at any length of time after RP (including more than 3 years after RP), and by restricting the cohort to patients with adverse pathological features (extracapsular extension, seminal vesical invasion, and/or positive surgical margins) to evaluate only those individuals eligible for ART. This study focused specifically on the cohort of patients who would be considered for ART, as the risk of complications has been shown to influence treatment decisions for such patients 6, and additional data could lead to better-informed choices. Both studies include propensity score adjustment, but with slightly different empiric assumptions in the statistical methodology. The difference in results between these two studies demonstrates the potential influence of investigators' methods in observational cohort studies and emphasizes a need for replicative and complementary studies in scientific inquiry.

In this study, IMRT was associated with a higher rate of urinary-incontinence complications, but not other urinary complications, compared to CRT. The reason for this finding is not clear, but a variety of factors could contribute to this finding. It is possible that trends toward higher RT doses over time have paralleled the observed trend toward increased IMRT utilization during the study period (from 33% in 2000–2004 to 78% in 2005–2007). This is a reasonable expectation based upon the publication of evidence during this time period that suggested a benefit to higher RT doses for post-RP RT 23,24. However, data regarding radiation dose and technical specifications are not available in the SEER-Medicare database, so the influence of dose cannot be evaluated in this study. In addition, detailed dosimetric characteristics of the IMRT and CRT plans and information on the definition of target volumes are not available. Consensus guidelines 9 for definition of the prostatic fossa were not published until after the designated study period, potentially leading to great variability in the definition of target volumes in this cohort. The volume of bladder receiving very high RT doses is predictive of GU toxicity 25,26, and IMRT can result in increased dose heterogeneity within the irradiated volume and an increased maximum bladder dose compared to 3D 10,11. It is not clear how variation in dosimetry could influence the observed results. One limitation of this analysis is that it is not possible to determine the etiology of urinary incontinence due to the combination of RP and RT, both of which could contribute to this complication. Baseline incontinence after RP could either improve or resolve prior to RT, thus the presence of baseline incontinence could not be adjusted for in our analysis. Although an explanation is not immediately available, the observed increase in urinary incontinence with IMRT, compared to CRT, after RP suggests a need for further evaluation in future studies.

Although no difference was observed between IMRT and CRT for procedures for ED, parameters that were associated with increased risk of ED included minimally invasive RP (vs. open RP), Hispanic origin, and time interval from RP to RT. That RT delivered within 90 days of RP, compared to more later time intervals, was associated with decreased risk of ED procedures is a surprising observation, and contradicts the clinical practice of delaying RT as long as possible to reduce risk of ED after RP 5. Although provocative, this finding should be interpreted with caution, as data are not available regarding baseline sexual function and patient motivation for interventions, which may influence whether procedures are performed 27.

The strengths of this study include the large size of the cohort and the evaluation of outcomes in a real-world, noncontrolled setting. However, there are limitations related to the use of the SEER-Medicare data to evaluate outcomes after RT in this setting 28,29. For instance, data are not available regarding several important elements that could potentially affect the risk of complications, including RT dose, doses delivered to bladder and rectum, the use of image guided radiation therapy (IGRT), and other technical details. There is no information for the fields used for RT, so it is possible that the RT delivered was in fact not to the prostate fossa, but to another body site such as bone in the setting of metastatic disease. In the current analysis, we addressed this issue by excluding those subjects who received RT for a diagnosis of bone metastasis in the absence of a prostate cancer code. However, it is possible that some subjects in our cohort received RT for bone metastasis rather than for the prostate bed. The presence of the bone metastasis code (198.5) was rare in our cohort, recorded for only seven subjects at the start of their RT. In the definitive treatment of patients with intact prostate glands, there are data to suggest that image-guidance approaches during RT delivery are associated with a decreased rate of grade 2+ GU toxicity at 2 years (10% vs. 20%), when compared to treatment without image-guidance 30. Localization of the target volume on pretreatment imaging allows for decreased planning target volume margins and potentially improved avoidance of critical structures over the course of treatment. Furthermore, the available details are limited regarding post-RT complications, so it is possible that the risk estimates can over-or underestimate the occurrence of events attributable to RT. Additionally, there is a difference in the duration of follow-up between the two study groups, and the observed complication rates could change with increased follow-up in the IMRT group. Therefore, it is possible that this study overestimates the comparative benefits of IMRT (vs. CRT).

A trend toward increased use of IMRT was observed during the study period, with 78% of subjects diagnosed between 2005 and 2007 receiving IMRT. This is similar to the rate reported recently by Goldin and colleagues in their analysis of SEER-Medicare data 22, as well as to the findings of a national survey of radiation oncologists regarding their practice policies for post-RP RT 6. It is critical that the potential advantages of IMRT be weighed carefully in light of the added costs of advanced technologies 12. In light of the mixed results observed with IMRT in this study, and the lack of differences observed by Goldin et al. in their analysis 22, additional research should be pursued to more thoroughly evaluate the role of IMRT after RP and to determine whether IMRT offers value in this setting.

## Conclusions

The use of IMRT after prostatectomy was associated with decreased risk of GI complications, when compared to CRT, but an increased risk of urinary-incontinence complications. Although this observational cohort study provides some insights into outcomes after post-RP RT, additional studies are needed to evaluate the comparative effectiveness and cost-effectiveness of advanced RT technologies in this context.

## Conflict of Interest

None declared.

## Supporting Information

Figure S1 Cohort selection diagram for prostate cancer patients in SEER-Medicare database who received IMRT or CRT after prostatectomy. The analysis compared outcomes after IMRT (*n* = 634) and CRT (*n* = 1052). RP, radical prostatectomy; RT, radiation therapy; CRT, conformal radiation therapy; IMRT, intensity-modulated radiation therapy; Dx, diagnosis.

**Table S1** Diagnosis and procedure codes used to define treatment type and complications. ICD-9, International Classification of Diseases version 9; CPT, Current Procedural Terminology; HCPCS, Healthcare Common Procedural Coding System; MIRP, minimally invasive radical prostatectomy; ORP, open radical prostatectomy. **Table S2**. Time to first complication, by type, for subjects receiving CRT or IMRT. Time is defined from the start of radiation therapy. **Table S3.** Complication rates without adjustment by propensity score, defined by procedure and diagnosis codes, listed according to class of complications.
