# Supplementary material for: Treatment-related complications of radiation therapy after radical prostatectomy: comparative effectiveness of intensity-modulated versus conformal radiation therapy
Source: Cancer Med. 2014 Feb 12;3(2):397–405. doi: 10.1002/cam4.205 (PMC3987089; doi:10.1002/cam4.205)
Supplement: Supplementary file 2 — Table S1 Diagnosis and procedure codes used to define treatment type and complications. ICD-9, International Classification of Diseases version 9; CPT, Current Procedural Terminology; HCPCS, Healthcare Common Procedural Coding System; MIRP, minimally invasive radical prostatectomy; ORP, open radical prostatectomy. Table S2. Time to first complication, by type, for subjects receiving CRT or IMRT. Time is defined from the start of radiation therapy. Table S3. Complication rates without adjustment by propensity score, defined by procedure and diagnosis codes, listed according to class of complications. [file cam40003-0397-sd2.docx]

**Supplemental Table 1.** Diagnosis and procedure codes used to define treatment type and complications. ICD-9=International Classification of Diseases version 9; CPT=Current Procedural Terminology; HCPCS= Healthcare Common Procedural Coding System; MIRP=minimally-invasive radical prostatectomy; ORP=open radical prostatectomy

| **Variable** | **Diagnosis Codes** | **Procedure Codes** | |
| --- | --- | --- | --- |
|  | **ICD-9 Diagnostic Codes** | **ICD-9 Procedure Codes** | **CPT/HCPCS Procedure Codes** |
| **Treatment** |  |  |  |
| Radical Prostatectomy  MIRP  ORP | --  -- | --  60.5 | 55866  55810, 55812, 55815, 55840, 55842, 55845 |
| IMRT | -- | -- | 77301, 77418, 0073T |
| CRT | -- | 92.24, 92.26 | 77305, 77310, 77315, 77321, 77371, 77372, 77373, 77402, 77403, 77404, 77406, 77407, 77408, 77409, 77411, 77412, 77413, 77414, 77416, 77422, 77423 |
| **Complications** |  |  |  |
| Gastrointestinal | 555-558, 558.1, 560.81, 560.89, 560.9, 564.5, 565, 566, 569.2-569.4, 569.41, 569.8, 569.81, 578, 578.9, 787.91 | 45.23, 45.25, 46.03, 46.1x, 48.23, 48.24, 48.31-48.33, 48.4-48.7, 48.62, 48.73, 48.9, 48.93, 49.1, 49.21, 49.23, 49.6, 49.7, 49.73, 93.95, 96.22, 96.23 | 44140-44160, 44204-44212, 45000, 45005, 45020, 45110-45123, 45300-45345, 45355-45387, 45500, 45562, 45563, 45800-45805, 45820-45825, 45905, 45910, 46600-46615, 46700, 99183, G0167 |
| Genitourinary Incontinence | 596.1, 596.2, 599.1, 599.82, 788.3x | 59.3-59.6, 57.83, 57.84, 58.43, 58.93, 59.71, 59.72, 59.79, 89.21-89.25 | 44660, 44661, 51715, 51725, 51726, 51736, 51741, 51772, 51784, 51785, 51792, 51795, 51797, 51798, 51840, 51841, 53440, 53442, 53443, 53445, 53447, 53520 |
| Genitourinary non-incontinence | 595.82, 596.0, 596.7, 598.x, 599.6, 788.2x | 57.85, 57.91-57.93, 58.0, 58.1, 58.3x, 58.44, 58.46, 58.47, 58.5, 58.6, 58.99, 60.2x, 60.95 | 52275, 52276, 52281-52283, 52510, 52601, 52612, 52614, 52620, 52630, 53010, 53400, 53405, 53410, 53415, 53420, 53425, 53600, 53601, 53605, 53620, 53621, 53850, 53852 |
| Erectile dysfunction | 607.84 | 64.94-64.97 | 54231, 54235, 54400-54402, 54405-54411, 54415-54417, C1007, C1813, C2622, C3500, C8514, C8516, C8534, J0270, J0275, J2440, J2760, L7900 |
| Hip Fracture | 733.14, 733.97, 805.6, 805.7, 806.6, 806.7, 808, 820x | -- | -- |

**Supplemental Table 2.** Time to first complication, by type, for subjects receiving CRT or IMRT. Time is defined from the start of radiation therapy.

|  |  | **CRT** | | | **IMRT** | | |
| --- | --- | --- | --- | --- | --- | --- | --- |
|  |  | **Median*** | **(25th, 75th)*** | **2-year Complication Rate^** | **Median*** | **(25th, 75th)*** | **2-year Complication Rate^** |
| **GI** | **procedure** | 29.3 | (19.1, 44.4) | 22.8% | 22.4 | (15.9, 30.6) | 17.2% |
|  | **diagnosis** | 29.4 | (18.7, 47.4) | 20.6% | 22.0 | (14.8, 29.3) | 15.2% |
| **UI** | **procedure** | 36.2 | (20.5, 57.7) | 7.6% | 17.9 | (14.3, 25.0) | 18.0% |
|  | **diagnosis** | 28.9 | (17.0, 51.7) | 14.0% | 16.6 | (13.9, 22.7) | 23.1% |
| **UN** | **procedure** | 36.4 | (19.4, 58.0) | 6.1% | 19.9 | (14.4, 30.6) | 5.9% |
|  | **diagnosis** | 32.4 | (18.9, 53.0) | 12.1% | 18.8 | (14.5, 27.5) | 15.5% |
| **ED** | **procedure** | 24.7 | (16.7, 37.0) | 2.0% | 19.9 | (15.6, 22.8) | 2.2% |
|  | **diagnosis** | 22.0 | (15.7, 38.3) | 15.8% | 16.6 | (13.8, 23.3) | 19.9% |
| *****Among the subset of patients with a complication | | | | |  |  |  |
| ^Uses all patients and incorporates censoring | | | |  |  |  |  |

GI = gastrointestinal; UI = urinary incontinence, UN = urinary nonincontinence; ED = erectile dysfunction

**Supplemental Table 3.** Complication rates without adjustment by propensity score, defined by procedure and diagnosis codes, listed according to class of complications.

|  | **CRT**  (n = 1,052) | **IMRT**  (n = 634) | **IMRT vs. CRT Comparison** |
| --- | --- | --- | --- |
| **Complications by Class** | **Events/100 person years** | **Events/100 person years** | **Rate Ratio**  **(95% CI)** |
| **Erectile Dysfunction**  Procedure  Diagnosis | 0.68  5.71 | 0.78  8.93 | 1.15 (0.59, 2.23)  1.56 (1.26, 1.95) |
| **Gastrointestinal**  Procedure  Diagnosis | 13.76  11.30 | 9.83  8.81 | 0.71 (0.59, 0.87)  0.78 (0.63, 0.96) |
| **Urinary – Incontinence**  Procedure  Diagnosis | 4.30  6.86 | 8.13  10.53 | 1.89 (1.50, 2.38)  1.53 (1.25, 1.88) |
| **Urinary – Non-incontinence**  Procedure  Diagnosis | 3.22  6.45 | 3.20  7.72 | 0.99 (0.71, 1.38)  1.20 (0.96, 1.50) |
