# Supplementary material for: Treatment-related complications of radiation therapy after radical prostatectomy: comparative effectiveness of intensity-modulated versus conformal radiation therapy
Source: Cancer Med. 2014 Feb 12;3(2):397–405. doi: 10.1002/cam4.205 (PMC3987089; doi:10.1002/cam4.205)
Supplement: Supplementary file 2 [file cam40003-0397-sd1.docx]

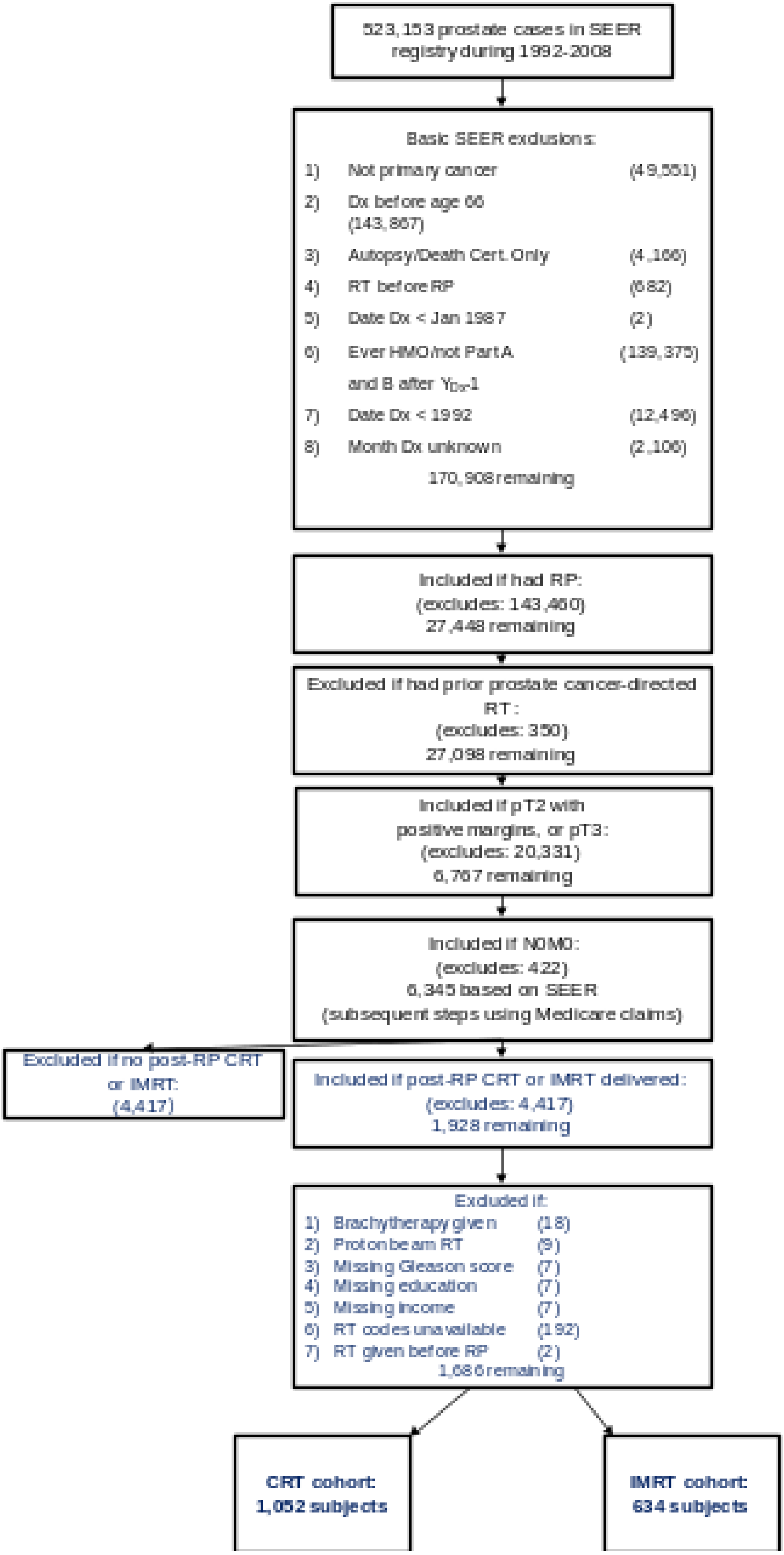
523,153 prostate cases in SEER registry during 1992-2008
Basic SEER exclusions:
1) Not primary cancer (49,551)
2) Dx before age 66 (143,867)
3) Autopsy/Death Cert. Only (4,166)
4) RT before RP (682)
5) Date Dx < Jan 1987 (2)
6) Ever HMO/not Part A and B after Y_{Dx}-1 (139,375)
7) Date Dx < 1992 (12,496)
8) Month Dx unknown (2,106)
170,908 remaining
Included if had RP:
(excludes: 143,460)
27,448 remaining
Excluded if had prior prostate cancer-directed RT:
(excludes: 350)
27,098 remaining
Included if pT2 with positive margins, or pT3:
(excludes: 20,331)
6,767 remaining
Included if N0M0:
(excludes: 422)
6,345 based on SEER
(subsequent steps using Medicare claims)
Excluded if no post-RP CRT or IMRT:
(4,417)
Included if post-RP CRT or IMRT delivered:
(excludes: 4,417)
1,928 remaining
Excluded if:
1) Brachytherapy given (18)
2) Proton beam RT (9)
3) Missing Gleason score (7)
4) Missing education (7)
5) Missing income (7)
6) RT codes unavailable (192)
7) RT given before RP (2)
1,686 remaining
CRT cohort: 1,052 subjects
IMRT cohort: 634 subjects
